# Molecular analysis of swine hepatitis E virus from north India

**Published:** 2010-11

**Authors:** Nargis Begum, Sunil K. Polipalli, Syed A. Husain, Premashis Kar

**Affiliations:** * *Department of Medicine, Maulana Azad Medical College, University of Delhi*; ** *Department of Biosciences Jamia Millia Islamia, New Delhi, India*

**Keywords:** Genotype, hepatitis E virus, phylogenetic analysis, swine

## Abstract

**Background & objectives:**

Hepatitis E is the main cause of enterically transmitted non-A, non-B hepatitis in developing countries. In the developed countries such as the USA, Japan and Taiwan, the viruses infecting humans and swine share the same genotype with a high sequence similarity. Genotype 1 circulates in humans whereas genotype 4 in pigs in India. The present study was designed to investigate the presence of anti-HEV antibodies and HEV-RNA in swine population from north India, to investigate the genotype prevalent in it, and to compare it with other swine and human HEV strains from India.

**Methods:**

A total of 67 serum samples were collected from pigs of age period (1-6 months) from Indian Veterinary Research Institute (IVRI), Izatnagar, Bareily and subjected to anti-HEV IgG and HEV RNA detection. A phylogenetic tree was constructed using the neighbor-joining method and evaluated using the interior branch test method with MEGA 4 software.

**Results:**

Anti-HEV IgG and HEV RNA was found in 38.8 and 4.5 per cent of swine samples studied respectively. The above samples were observed to be of genotype 4e. The three new sequences had nucleotide similarity with other swine sequences in genotype 4 ranging from 80-98 per cent.

**Interpretation & conclusions:**

The three sequences observed in the present study showed nucleotide similarity with other swine sequences from southern and western India. The present study suggests that genotype 4 ‘e’ is prevalent in the north India.

Hepatitis E is the main cause of enterically transmitted non-A, non-B hepatitis in developing countries. Hepatitis E virus (HEV) is a member of the genus Hepevirus. It is a non-enveloped, single-stranded RNA virus of approximately 7.2 kb in length^1^. Its genome is encoded by 3 separate but partially overlapping open reading frames (ORFs)^2^. ORF1 likely encodes non-structural viral proteins, ORF2 encodes the putative capsid protein and ORF3 encodes a cytoskeleton-associated phosphoprotein^3^–^5^. Four major genotypes of mammalian HEV have been identified on the basis of complete genome sequences^6^. Genotype 1 includes human isolates from Asia and North America, genotype 2 comprises human isolates from Mexico and some African countries, genotypes 3 and 4 include human and swine strains isolated in industrialized countries as well as developing areas.

HEV-RNA and antibodies to HEV have been found in a wide variety of animals, especially swine^7^–^10^. It was hypothesized that zoonosis was involved in the transmission of HEV, especially for the cases in non-endemic areas. Studies by Meng *et al*^11^–^13^ provided initial evidence for the possibility of such spread in US. Subsequently, circulation of swine HEV was documented in several countries such as Taiwan^14^, Japan^15^, The Netherlands^16^, Canada^17^ and India^18^.

In countries such as the USA, Japan and Taiwan, the viruses infecting humans and swine share the same genotype with a high sequence similarity^14^–^16^. However, studies from India reported that genotype 1 circulates in humans whereas genotype 4 in pigs^18^–^20^. The aim of the present study was to investigate the presence of anti-HEV antibodies and HEV-RNA in swine population from north India, to investigate the genotype prevalent in swine, and to compare it with other swine and human HEV strains from India and different areas of the world.

## Material & Methods

*Samples*: A total of 67 serum samples were collected from pigs of age period (1-6 months) from Indian Veterinary Research Institute (IVRI), Bareily, India in July 2005. The serum samples were stored at -40°C until tested for anti-HEV IgG and HEV RNA.

*ELISA for anti-HEV IgG*: All serum samples were thawed at room temperature and tested with IgG anti-HEV ELISA kits (Genelabs Diagnostics, Singapore). This commercially available assay is based on the ORF2 and ORF3 recombinant proteins of the Burmese and Mexican strains of HEV. The ELISA was performed according to the protocols provided by the manufacturer. All the samples were assayed in duplicate.

*RNA extraction and reverse transcription polymerase chain reaction*: RNA was extracted from 100 µl of serum sample by using TRIZOL reagent (Invitrogen, USA) in accordance with the manufacturer’s protocol in PCR Hepatitis Lab, Department of Medicine, Maulana Azad Medical College, New Delhi. The viral RNA was finally dissolved in 20 µl Rnase-free water. The nested PCR was performed in all the samples using primers for genotypes 1 and 4, as these two genotypes have been reported from India^18^–^21^. The primers used for genotype 1 were external sense: 5'- CCG GAT CCA CAC ACA TCT GAG CTA CAT TCG TGA GCT- 3', external anti-sense: 5'- CCG AAT TCA AAG GCA TCC ATG GTG TTT GAG AAT GAC- 3', internal sense: 5'- GGA ATT CGA CTC CAC CCA GAA TTA CTT- 3', and internal anti-sense 5'- GGA ATT CAC AGC CGG CGA TCA GGA CAG- 3'. These two sets of primers were designed to produce 343 bp segment of ORF1 region^21^. The primers used for genotype 4 were external sense: 5'- AAT ACA CCT TAC ACT GGC GCC CT- 3', external anti-sense: 5'- TCA GCA AGA TTA AAT AAG GTC AGC GC- 3', internal sense: 5'- ACA CTG GCG CCC TCG GTC TGC T- 3', and internal anti-sense 5'- AGA TTA AAT AAG GTC AGC GCT ATA CCA C- 3'. These two sets of primers were designed to produce 246 bp segment of ORF2 region^18^.

The parameters for first-round PCR for both the sets of primers for genotypes 1 and 4 included initial denaturation 95°C for 5 min, followed by 35 cycles of denaturation for 1 min at 94°C, annealing for 1 min at 50°C, extension for 1 min at 72°C and a final incubation at 72°C for 7 min. The parameters for the second-round PCR were similar.

*Nucleotide sequencing and phylogenetic analysis*: The nested PCR products were purified using QIA quick PCR purification Kit (QIAGEN, Inc. Germany). The purified DNA was subjected to direct sequencing. The 220-nt consensus sequences were aligned using CLUSTAL W programme and phylogenetic tree was constructed using the neighbor-joining method and evaluated using the interior branch test method with MEGA 4 software^22^. Prototype HEV strains used as references in the analysis and their Gen Bank accession numbers are shown in the Fig. The sequences determined in this study were deposited in Gen Bank database under the accession numbers EU003603, EU003604 and EU003605.

**Fig:**
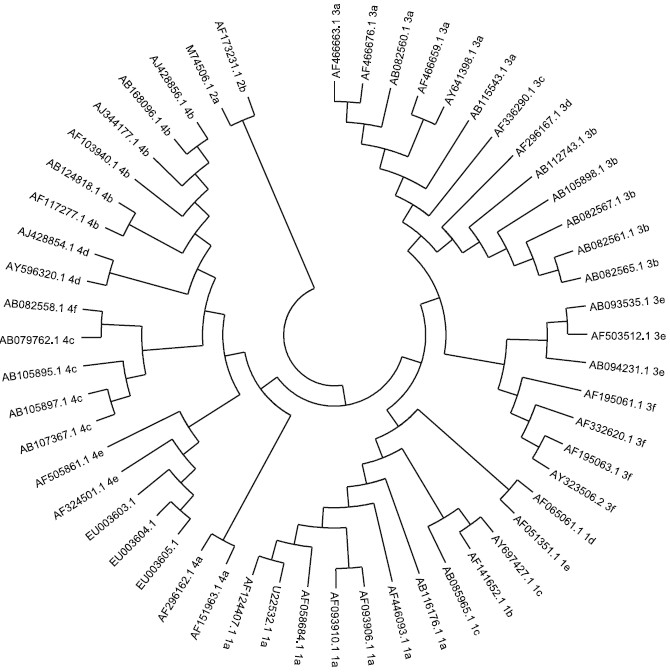
Phylogenetic relationship among swine and human strains of hepatitis E virus (HEV) representing the 4 major genotypes, based on 230 nucleotide fragment of ORF2 of the genome.

The study protocol was approved by the Institute Animal Ethical Committee of Maulana Azad Medical College, New Delhi.

## Results

Of the 67 samples tested for IgG anti-HEV in the first round of ELISA, 26 samples were positive and 3 samples were in gray zone/cut-off index, however, when the test was repeated, these three samples showed negative results. Therefore, the overall presence of anti-HEV IgG in swine samples tested was found to be 38.8 per cent.

Using the primers for genotype 1, none of the 67 samples showed the presence of HEV RNA, which suggested absence of HEV genotype 1 infection among these swine samples. However, when the primers of genotype 4 were used, three samples (4.5%) showed the presence of HEV RNA. The occurrence of IgG anti-HEV and HEV RNA in the serum samples of swine at different months of age is shown in Table I. The serum samples positive for HEV RNA were also positive for anti-HEV IgG.

**Table I:** Detection of IgG anti-HEV and HEV RNA in serum samples of pigs of various ages

Age (months)	No. of samples	IgG anti-HEV N (%)	HEV RNA N (%)
1-3	13	3 (23.1)	2 (5.7)
3-5	19	8 (42.1)	1 (5.3)
>5	35	15 (42.8)	0 (0)
Total	67	26 (38.8)	3 (4.5)

The amplified PCR product was confirmed to be of HEV strain by direct sequence analysis using the BLAST programme. Partial sequences of 230 bp of HEV ORF2 were compared with others from the known genotypes and were found to cluster in genotype 4e group. The three sequences of the present study had nucleotide similarity (Table II) with other swine sequences in genotype 4 ranging from 80.6-97.8 per cent^7^,^18^,^19^. The closest relationship (87.3%) between these sequences and human strains in genotype 4 was between HEVS and Chinese and Japan human isolates. All the sequences formed single clustered of subgroup ‘e’ in the genotype 4.

**Table II:** Nucleotide similarity of 220-base-pair fragment of open reading frame 2 of the HEV genome of swine and human strains of different genotypes, as compared with 3 strains isolated in this study

Type	Isolate	Country	Host	Nucleotide identity per cent (%)		
				HEVS	HEVS1	HEVS2
4e*	EU003605	India (North)	Pig		97	97
4e*	EU003604	India (North)	Pig	97	-	98.3
4e*	EU003603	India (North)	Pig	97	98.3	-
1a	AF124407	India	Human	72.8	72.6	71.6
1a	AF446093	India	Human	74.6	74.3	73.3
1a	AF058684	Spain	Sewage	73.2	73	72
1a	AF093906	India	Human	72.8	72.6	71.6
1a	U22532	India	Human	74.1	73.9	72.8
1a	AB116176	Nepal	Human	72.4	72.2	71.1
1b	AF141652	China	Human	74.6	74.3	73.3
1c	AB085965	Nepal	Human	73.7	74.8	73.7
1c	AY697427	Kyrgyzstan	Human	74.6	74.8	73.7
1d	AF065061	Morocco	Human	73.2	73	72
1e	AF051351	Egypt	Human	72.8	71.7	70.7
2a	M74506	Mexico	Human	72.0	71.8	70.8
2b	AF173231	Nigeria	Human	44.5	46	45.1
3a	AF466676	US	Pig	67.2	66.8	67.2
3a	AF466663	US	Pig	75.7	73.7	73.5
3a	AB082560	Japan	Human	77.2	75.2	75
3a	AB115543	Japan	Human	68.9	68	68.5
3a	AY641398	Korea	Human	75	73.9	72.8
3a	AF466667	US	Pig	77	74.6	74.8
3b	AB082567	Japan	Human	73.7	72.6	71.6
3b	AB112743	Japan	Human	68	67.2	67.6
3b	AB105898	Japan	Pig	74.3	72.8	71.8
3c	AF336290	Netherlands	Pig	67.9	67.9	68.7
3d	AF296167	Taiwan	Pig	72.2	71.1	70.1
3e	AF503512	UK	Pig	69.3	67.5	68.9
3e	AB093535	Japan	Human	67.6	66.7	67.2
3e	AB094231	Japan	Pig	67.6	66.7	67.2
3f	AF332620	Netherlands	Pig	68.9	67.6	68.5
3f	AF195061	Spain	Human	68.9	68.9	68.5
3f	AF195063	Spain	Human	69.7	68.7	69.3
3f	AY323506	Spain	Pig	68.9	68.5	68.9
4a	AF151963	China	Human	83.8	83	82.3
4a	AF296162	Taiwan	Human	81.6	80.9	80.2
4b	AJ344177	China	Human	87.3	86.5	84.9
4b	AB168096	Japan	Human	87.3	85.7	84.9
4b	AF103940	China	Human	87.3	85.7	84.9
4b	AB124818	Indonesia	Pig	85.1	84.3	82.8
4b	AJ428856	China	Pig	86.4	84.8	84.1
4b	AF117277	Taiwan	Human	86	84.3	83.6
4c	AB105895	Japan	Human	84.2	83.5	82.8
4c	AB107367	Japan	Human	84.2	83.5	82.8
4c	AB079762	Japan	Human	82.9	81.3	80.6
4c	AB105897	Japan	Human	84.6	83.9	83.2
4d	AY596320	China	Pig	81.1	82.2	80.6
4d	AJ428854	China	Pig	83.3	84.3	82.8
4e	AF324501	India (West)	Pig	97.4	97.8	96.1
4e	AF505861	India (South)	Pig	93.9	91.7	90.9
4f	AB082558	Japan	Human	83.3	81.7	81

* Present study.

*Source*: The nucleotide sequences of above HEV isolates were retrieved from Genbank

## Discussion

Anti-HEV antibodies have been shown among pigs and other animals in several HEV-endemic and non-endemic countries, including India^7^,^8^,^10^. Pigs stand out as being an animal group with the highest rate of anti-HEV seropositivity. In the present study, the anti-HEV IgG positivity (38.8%) among pigs was somewhat lower than 43-74.4 per cent (western & south India) and 97.5 per cent (Lucknow) reported previously among Indian pigs^10^,^18^,^19^. This may be due to the difference in age of pigs at which the samples have been drawn. In the present study, it appears that anti-HEV IgG positivity increased with increasing age of pigs.

Serum samples from pigs older than 5 months were tested negative, similar to the study from Lucknow^10^, which showed presence of HEV RNA in only one of the 200 serum samples collected from adult pigs. However, infection with HEV is associated with a short time of detectable HEV RNA in serum, which is followed by development of anti-HEV antibodies that may last for a long time and pre-existing anti-HEV IgG can prevent HEV viraemia^23^. Thus, detection of HEV RNA is less likely in older pigs than in the younger^24^,^25^.

The strongest evidence in favour of animal-to-human transmission of a pathogen is provided by an identity or close resemblance of isolates from these sources. Swine-to-human transmission hypothesis for HEV was supported by accumulated evidences^26^–^29^. The most direct evidence of animal-to-human transmission of HEV came from Japan, where four human cases of hepatitis E were linked to the consumption of uncooked deer meat, based on 99.7-100 per cent nucleotide sequence homology between the virus recovered from patients and the left-over meat^15^. In the present study, the three sequences were phylogenetically related to the genotype 4 and shared 71.6-74.6 per cent homology with human isolates of India, based on the partial ORF2 sequences. The sequences showed least nucleotide homology with genotype 2 ‘b’ which ranged from 44.5-46 per cent.

In the present study, the strains phylogenetically clustered into genotype 4 and formed single subgroup ‘e’, sharing 90.9-97.8 per cent homology with inter-subgroup and 80.2-83.8, 82.8-87.3, 80.6-84.6, 80.6-84.3, and 81-83.3 per cent intra-subgroup identity homology with subgroup ‘a’, ‘b’, ‘c’, ‘d’ and ‘f’ respectively. Moreover, the present sequences showed 80.2-81.9 per cent homology with the only swine sequence reported from Lucknow^19^. This suggests that in north India, different subgroups may be present; such high difference between the nucleotide identities of swine sequences is not observed in west and south India.

In conclusion, the study confirms the circulation of genotype 4 ‘e’ in swine from north India similar to southern and western India suggesting genotype 4 ‘e’ to be predominant in Indian pigs. Other subgroups may also be present which can only be identified by sequencing more samples from north India.
